# Keeping the Faith: Religion, Positive Coping, and Mental Health of Caregivers During COVID-19

**DOI:** 10.3389/fpsyg.2021.805019

**Published:** 2022-01-20

**Authors:** Heera Elize Sen, Laura Colucci, Dillon T. Browne

**Affiliations:** Department of Psychology, University of Waterloo, Waterloo, ON, Canada

**Keywords:** religion and spirituality, coping, disaster, COVID-19, mental health

## Abstract

The COVID-19 pandemic has resulted in major stressors such as unemployment, financial insecurity, sickness, separation from family members, and isolation for much of the world population. These stressors have been linked to mental health difficulties for parents and caregivers. Religion and spirituality (R/S), on the other hand, is often viewed as promotive of mental health. However, the mechanisms by which R/S might promote mental health for parents during the pandemic remain unclear. Thus, this longitudinal study explores how R/S is associated with better caregiver mental health during the COVID-19 pandemic through higher levels of positive coping skills. A sample of *N* = 549 caregivers (parents and other adults in childrearing roles) across Canada, the United States, the United Kingdom, and Australia were recruited through the Prolific^®^ research panel [67.8% female; age *M* = 41.33 years (*SD* = 6.33), 72.3% White/European]. Participants were assessed on measures of psychological distress, coping, R/S, and COVID-19 disruption at three time points between May and November 2020. Cross-lagged panel analysis revealed that caregiver coping mediated the relationship between caregiver R/S and caregiver mental health. Findings highlight a mechanism through which R/S naturally conveys a mental health benefit during periods of social disruption, which may provide an important target for public health promotion and clinical intervention.

## Introduction

The COVID-19 pandemic has resulted in unprecedented social disruption, including prolonged isolation and loss of physical contact, financial insecurity, marked change in routines, and a fundamental shift to family life for billions around the world. These major stressors have resulted in widespread emotional distress and increased prevalence of mental illness (Cooke et al., 2020). Indeed, stressors emerging from the COVID-19 pandemic are associated with increased psychological distress, anxiety, and depression for young people (Li and Leung, 2020; Singh et al., 2020; Browne et al., 2021b), parents and other caregivers of children (Wade et al., 2021), and the general population (Xiong et al., 2020). Disasters can deprive and drain one’s personal and mental resources, and the imposition of public health precautions can result in a dwindling of one’s network of support (Aten et al., 2015). As additional waves of the pandemic surge and new stressors continue to emerge, individuals and families are forced to mobilize their coping resources. It is possible that religion and spirituality (R/S) may offer a unique source of strength during this difficult time, thereby enhancing coping and, consequently, mental health (Walsh, 2020).

The significant mental health burdens experienced by parents and caregivers (i.e., kin and non-kin adults responsible for raising children) during natural and human-made disasters are well-documented (North, 2016). As of this writing (December 2021), the putative consequences of the COVID-19 pandemic, specifically for parents and families, are already well established (Patrick et al., 2020; Russell et al., 2020; Spinelli et al., 2021). Replicated observations of mental health consequences for caregivers during COVID-19 underscore the importance of studying factors that can help promote better mental health during disasters. However, there is a relative paucity of COVID-19 family process literature articulating the factors and mechanisms that correspond to better mental health and family functioning, which are not simply the absence of risk factors (Walsh, 2020). Furthermore, the importance of studying factors that promote caregiver mental health is necessitated by the observance of spillover effects from caregivers to children (Katz and Gottman, 1996) and the family systems framework that suggests that every individual in the family is influenced by other members (Steinglass, 1987). In this way, pandemic stressors for adults have the potential to impede children’s mental health and development, as well (Prime et al., 2020; Pitchik et al., 2021). Thus, it is imperative to also study factors that support and enhance caregiver functioning, as these mechanisms may analogously spillover to convey advantage across the family unit, thereby offsetting the marked challenges so many families are facing today.

### Religion and Spirituality During Disasters

Religion and spirituality should be studied as a factor that relates to mental health during disasters due to (a) the overwhelming presence of religious and spiritual beliefs and practices around the world and (b) the capacity for religious and spiritual beliefs to provide a powerful lens through which individuals and families can make meaning of the events that occur in their lives (Cherry et al., 2015; Aten et al., 2019; Walsh, 2020). In an international random sample, the Haerpfer et al. (2020) found that an overwhelming 71.1% (*n* = 70,867) of participants reported that religion was “rather important” or “very important” in their lives. Furthermore, 62.9% of those surveyed explicitly identified as being religious (World Values Survey, 2020). Individuals have long turned to R/S to make meaning of and cope with disasters (Marks et al., 2009) and family science—in its multi-level conceptualizations of well-being—has considered R/S to be promotive of mental health and subjective well-being amongst caregivers (Marks and Dollahite, 2016; Walsh, 2016b,^2020^; Browne et al., 2021a,b). Disasters take away a sense of normalcy and disrupt one’s daily routine; the beliefs, practices, and institutions and practices associated with R/S can provide a familiar structure and help frame negative events in a positive light, thereby helping individuals and caregivers through these kinds of overwhelming and unprecedented stressors.

The beliefs and practices associated with R/S can be a powerful source of hope and affirmation. However, they can also be (at times) a source of rejection and denigration (Exline et al., 2000). Research on the relationship between R/S and mental health outcomes appears to be conflicted (e.g., Furnham and Brown, 1992; Plante and Canchola, 2004; Rose et al., 2015). The beliefs and dogmas associated with religious and spiritual communities can increase their members’ risks for mental and emotional distress if they correspond to negative (unhealthy, unhelpful) coping styles. Negative coping associated with R/S can include religious or spiritual appraisals of an event as being a punishment from their deity (or deities in polytheistic religions) for being insufficiently devout or questioning the power and strength of their R/S (Pargament et al., 2011). However, positive religious coping, when compared with other ways of coping, appears to be especially helpful in situations such as bereavement or serious illness, where little direct control is possible (Koenig, 2012). During extreme and uncontrollable stressors such as disasters, an individual’s perceived support from their deity/deities or members of their religious congregation may reduce the deleterious outcomes of the stressors due to enhanced coping. Stated differently, R/S beliefs could provide the psychological foundations that promote mental health amongst caregivers amidst an event that is largely out of their control (Milstein et al., 2010).

### Positive Coping as a Mechanism of Religion and Spirituality

Connor and Davidson (2003) describe an individual’s degree of positive “stress coping ability” (p. 1) as an important mechanism in the emergence of resilience. In the developmental and family literature, resilience is most often defined as a process through which individuals or families experience adaptation despite adversity, usually through the presence of a protective factor that offsets this risk (Luthar et al., 2000), such as positive coping ability (Walsh, 2016a,b). This terminology is valuable as it reflects an understanding of resilience as a process that results in well-being and mental health. This contrasts with the erroneous trait-based conceptualizations of resilience that may indirectly place blame on individuals who struggle to cope adaptively amidst adversity (Luthar, 2015). Thus, positive coping reflects a core construct in the study of resilience, which is why it was considered as a primary mechanism linking R/S and mental health in the current study. The onslaught of stress brought about by disasters such as the pandemic should, theoretically, impair one’s mental health. Yet, we observe that many people tend to demonstrate adaptation (Frankl, 1992; Walsh, 2020). This pattern can be attributed to promotive factors such as social or familial support, or empathetic and emotionally responsive caregiving (Kumpfer, 2002; Zimmerman et al., 2013; Hostinar and Miller, 2019). In the same way, R/S and positive coping may operate as promotive factors that contribute to positive outcomes during times of global challenge, regardless of levels of risk (Hostinar and Miller, 2019).

The psychological benefits that may be conferred by R/S, align well with the tenets of the family resilience framework (a prominent conceptual model in family science and adversity research; Walsh, 2016b). Walsh’s model proposes that *making meaning of adversity*, *a positive outlook*, and *flexibility*, are three cognitive mechanisms through which families can bolster resilience and positive outcomes for all family members. R/S inherently encourages and fosters these processes, supporting meaning making by treating hardship as a normal part of life, and perhaps even a welcome occurrence that serves to fuel personal development. Human capacities for transforming pain into moments of growth provide a frame of reference for pure pain and loss, allowing suffering to be held in concert with meaning and hope (Frankl, 1992). Relatedly, Kierkegaard (2012) described how religious individuals possess a quality that he described as “epistemic flexibility” which is characterized by the ability to view any event as being subject to the “transformational power of God” or the ability to change any bad event into a “good and perfect” event. This epistemic flexibility, is (in its essence) a form of meaning making, which allows the perception of disappointments and suffering to be viewed as opportunities for God’s subsequent redemption. Simply put, meaning making of pain and suffering may be a core contributor to resilience and this perspective has garnered widespread attention in broader coping and adversity literature (Affleck et al., 1985; Yalom and Lieberman, 1991; Tedeschi and Calhoun, 2004; Ryff, 2014).

Additionally, R/S also encourages a positive outlook by reiterating the idea of hope. Most, if not all, forms of R/S emphasize the idea of “keeping the faith” despite any number of obstacles one might encounter, while simultaneously supporting learned optimism (Snyder et al., 2002). Religious institutions that ask believers to have faith in the idea that “everything happens for a reason” and that there is “a greater unknown plan” are well-positioned to have nurtured a mindset of finding meaning and hope amidst crises (Aten et al., 2019; Davis et al., 2019b). For example, Davis et al. (2019a) found that theistic survivors of Hurricane Katrina overwhelmingly perceived God as a “wise and benevolent guide” that would provide them with safe-haven. Similarly, theistic survivors of the Louisiana flood were found to engage in meaning-making following the disaster by drawing on representations of a “benevolent God’s providence” to appraise why the disaster might have occurred and what purpose it might serve in their lives (Davis et al., 2019a). Finally, R/S may help foster flexibility during disasters by providing a stable, grounding perspective due to the existence of an external authority who “has a plan” for the individual and family. Religious institutions provide a “grounding effect” during disasters and other traumatic events by providing a vantage point for individuals to create meaning of seemingly meaningless pain and suffering (Milstein, 2019). Thus, R/S, can promote they key positive components of coping, as defined by Walsh (2016a; 2016b). As discussed earlier, R/S’s ability to support mental health hinges on whether an individual’s beliefs and practices foster adaptive or maladaptive coping styles. This leads to the hypothesis that if R/S beliefs and practices support an individual’s adaptive coping styles, then it should serve as a mechanism (i.e., a mediator) through which R/S relates to better mental health outcomes over time.

### The Present Study: Overview and Hypotheses

The current study was purposed to examine the relation between R/S and caregiver mental health during COVID-19. Specifically, it was hypothesized that positive coping ability may operate as the psychological mediating mechanism through which R/S promotes mental health benefits and optimizes caregiver well-being. This area of inquiry was evaluated amongst a longitudinal sample of caregivers assessed during the COVID-19 pandemic (May–November 2020), while controlling for the severity of pandemic disruption for families. Through the employment of a cross-lagged panel model, it was possible to test a directional cascade, beginning with R/S, which predicted subsequent levels of coping and, later, mental health. The opposite direction of effects was not hypothesized. That is, we did not expect to see that caregivers with higher levels of mental health would have better coping and, subsequently, higher levels of R/S. In other words, individual differences in R/S were expected to instantiate a directional pathway which led to better coping and mental health outcomes as the pandemic unfolded.

## Materials and Methods

### Participants and Procedure

Participants come from the *Child Resilience and Managing Pandemic Emotional Distress in Families* (CRAMPED) Study, which is a longitudinal cohort designed to explore family dynamics during the COVID-19 pandemic. The dataset consists of a longitudinal cohort of target parents and caregivers with at least two children, as the current sample is part of a larger program of research concerned with examining within-family (i.e., sibling) dynamics. For eligibility, caregivers were defined in the study as adults who had child rearing responsibilities of minors who lived at least some of the time in the same house. Data collection was facilitated by the Prolific^®^ research panel across four different countries: Australia, Canada, the United Kingdom, and the United States. Prolific is a company that allows for the recruitment of online participants for research studies and permits the targeting of specific populations. Multiple countries were included in this study to maximize the sample size available through the Prolific platform. Exclusion criteria included people who (1) were not caregivers/parents, (2) had less than two children living with them at least some of the time, (3) had children under 5 years of age or over 18 years of age, and (4) had children not currently residing in the same household.

The present study is based on data collected at the initial baseline assessment (T1) in May 2020, and follow-up assessments held in September (T2), and November (T3) 2020. A sample of *N* = 549 caregivers (372 females, 158 males, 19 chose not to report sex) ranging in age from 24 to 62 (*M* = 41.33, *SD* = 6.33) was recruited. Ninety percent of the caregivers in this study were biological parents (only one per family). The remaining caregivers were parents of children by marriage/common law union, parents of adopted children and caregivers in extended family/kinship arrangements (e.g., a grandparent in a caregiver role). Additional descriptive statistics on the sample are presented in Table 1. Online consent and completion of surveys was facilitated via Qualtrics. All participants received information regarding the purpose, risks, and benefits of study participation in accordance with the criteria set out by the University of Waterloo’s ethics review board (Protocol # 42112).

**TABLE 1:** Descriptive statistics for study participants.

	Caregivers (*N* = 549)	
	*n*	*M (SD)*
**Age**	527	41.33 (6.33)
	***n***	**%**
**Sex**		
Female	372	67.8
Male	158	28.8
**Ethnicity**		
Asian–South	17	3.1
Asian–South East	9	1.6
Asian–East	2	0.4
Black–African	12	2.2
Black–North American	6	1.1
Black–Caribbean	1	0.2
Mixed Heritage	12	2.2
White–European	336	61.2
White–North American	61	11.1
Other	13	2.4

### Measures

#### COVID-19 Family Stressor Scale

The COVID-19 Family Stressor Scale (CoFaSS) was used to assess stressors and disruptions experienced by caregivers due to the COVID-19 pandemic (Prime et al., 2020). This measure was administered at the study baseline in May 2020. Participants responded to the question, “Since the COVID-19 disruption, have any of the following changes occurred in your household?”. The items in the CoFaSS included 16 options participants could select, such as “gone into financial debt,” “working from home while meeting family demands,” “experienced increased emotional withdrawal from family members,” and “separated from family or loved ones due to COVID-19.” Participants responded to each of the presented options on a three-point rating scale of how true the item was for them (1 = *not true*, 2 = *somewhat true*, 3 = *very true*), and a sum score of their responses was produced. Validation of the scale yielded three overarching dimensions (factors) of disruption, including financial, family/relational, and pandemic-specific stressors. The overall scale was utilized in the current study, and internal consistency was very good (α = 0.83).

#### Religiosity and Spirituality

Beliefs and practices associated with R/S were assessed using items from the Brief Multidimensional Measure of Religiousness/Spirituality (Milstein, 2019). Participants completed nine items surrounding religious beliefs on a five-point rating scale (1 = *strongly disagree* to 5 = *strongly agree*), four items on religious practices on a five-point rating scale (1 = *never* to 5 = *every day*), and one item on spirituality on a five-point rating scale (1 = *not at all* to 5 = *to a very great extent*). Internal consistency in the current sample was excellent (α = 0.96). Participants completed this measure at T1 and T3.

#### Positive Coping

Positive coping was assessed using an abbreviated version of the Connor-Davidson Resilience Scale (CD-RISC-10; Connor and Davidson, 2003; Campbell-Sills and Stein, 2007). The scale consisted of 10 items measuring positive stress coping ability (e.g., “I tend to bounce back after illness, injury, or other hardships” and “Having to cope with stress can make me stronger”). Items in this scale measure coping as a function of the following related factors: hardiness, social support/purpose, faith, and persistence (Campbell-Sills and Stein, 2007). Participants completed items on a five-point rating scale (1 = *not true at all* to 5 = *true nearly all the time*). Internal consistency in the current sample was excellent (α = 0.92). Participants completed this measure at T1, T2, and T3. We operationalized this tool specifically as a measure of positive coping, given that many items on this scale describe coping skills (e.g., “adapt to change,” “stay focused under pressure,” and “see humorous side of problems”). Additionally, our analysis is concerned with mediation, as R/S is viewed as a promotive factor that conveys a coping benefit regardless of the level of risk exposure (Hostinar and Miller, 2019). In contrast, studies of resilience as a process, typically utilize moderation (i.e., examining an interaction between a risk factor and protective factor; see Luthar et al., 2000).

#### Caregiver Psychological Distress

General mental health was measured using the Kessler 10 (K10), which is a 10-item measure of non-specific psychological distress used extensively in epidemiological settings (Kessler et al., 2003). The scale contains items that are most clinically related to symptoms of depression and anxiety. Participants completed items that measured feelings experienced in the last 30 days on a five-point rating scale (1 = *none of the time* to 5 = *all of the time*). For this study, the continuous version of the scale was selected. Internal consistency in the current sample was excellent (α = 0.93).

### Analyses

Preliminary analyses were conducted using IBM SPSS Version 27.0. Path analysis with a cross-lagged panel configuration was used to test the relationship between caregiver R/S beliefs and practices, positive coping, and psychological distress using AMOS 27.0. The sample size of this study is consistent the range that is typically expected within complex mediation analyses (Fritz and MacKinnon, 2007). Model fit was evaluated using three fit indices: the chi-square measure of overall goodness of fit, the comparative fit index (CFI), and the root mean square error of approximation (RMSEA; Hooper et al., 2008). Attrition over time and any other missing data was handled using Full Information Maximum Likelihood (FIML) estimation that is based upon a missing at random (MAR) assumption. FIML estimation allows for the maximum use of the data available as it neither substitutes values for missing data points nor deletes cases that have missing data points. The FIML method instead estimates parameter values that have the highest likelihood of being observed in the current sample data and then accumulates and maximizes that data. The significance of any indirect effects was evaluated using confidence intervals constructed using Monte Carlo simulation methods appropriate for structural equation models (Falk and Biesanz, 2016). Mediation was considered as significant if the 95% bias corrected and accelerated confidence intervals for the indirect effects did not include 0. All pathways controlled for caregiver age, sex, race (white or non-white participants), and pandemic-related disruption (measured using the CoFaSS) at T1.

## Results

**FIGURE 1:**
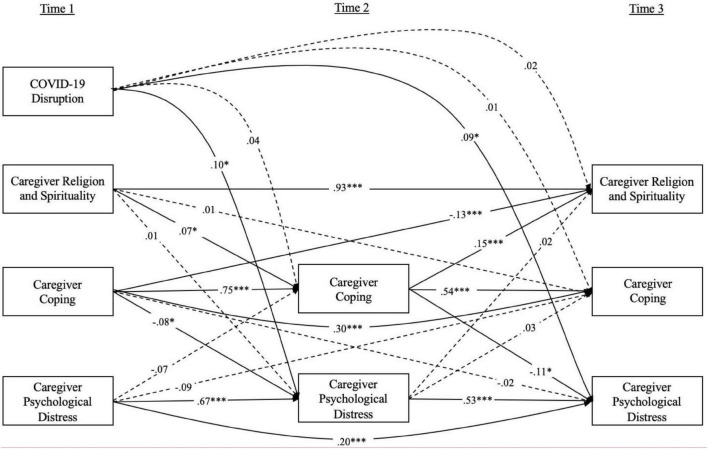
Cross-lagged regression model examining the proposed mediation model of caregiver resilience mediating the relationship between caregiver R/S and caregiver psychological distress. Values shown are standardized coefficients. Solid lines indicate significant parameter estimates. Dotted lines indicate non-significant parameter estimates. *p < 0.05, ***p < 0.001.

Bivariate correlations amongst all measures used in the mediation analyses are presented in Table 2. Figure 1 presents the cross-lagged panel model. The hypothesized model was fit to the data for the entire sample. The model closely fit the observed data: χ^2^(2) = 1.667, *p* = 0.435; CFI = 1.000; RMSEA = 0.000 [0.000, 0.080]. The standardized estimates of the full cross-lagged panel model are presented in Figure 1. Caregiver R/S beliefs and practices at T1 were positively associated with coping at T2, controlling for previous levels of coping. Furthermore, coping at T2 was inversely related to psychological distress at T3, controlling for previous levels of distress. Thus, there was a directional relationship where R/S in May predicted coping in September, which predicted mental health in November 2020, all in the hypothesized directions. Indirect effects analysis revealed a significant indirect effect of caregiver R/S beliefs and practices on caregiver psychological distress via caregiver coping (B = −0.0043, 95% CI = [−0.0109, −0.0000]).

**TABLE 2:** Bivariate correlations for measures included within mediation analyses.

	1	2	3	4	5	6	7	8	9
1. COVID-19 Family Stressor Scale (T1)	1								
2. Religiosity/Spirituality (T1)	0.047	1							
3. Kessler 10 (T1)	0.545*	–0.067	1						
4. Resilience (T1)	−0.197*	0.188*	−0.448*	1					
5. Kessler 10 (T2)	0.457*	–0.040	0.742*	−0.387*	1				
6. Resilience (T2)	−0.148*	0.192*	−0.388*	0.781*	−0.449*	1			
7. Kessler 10 (T3)	0.472*	–0.043	0.693*	−0.429*	0.765*	−0.446*	1		
8. Resilience (T3)	−0.194*	0.156*	−0.408*	0.753*	−0.396*	0.791*	−0.487*	1	
9. Religiosity/Spirituality (T3)	0.083	0.924*	−0.046*	0.156*	−0.047*	0.195*	–0.068	0.183*	1

*Correlation is significant at the 0.01 level (2-tailed).

To further strengthen conclusions surrounding the directional role of caregiver R/S at T1 predicting better mental health at T3 through more positive coping at T2, the opposite direction of associations was also considered in the context of the cross-lagged model (i.e., better caregiver mental health at T1 predicting more R/S at T3 via more positive coping at T2). Caregiver mental health at T1 was not significantly related to coping at T2, though coping at T2 did significantly predict higher levels of R/S at T3, and controlling for previous levels of these variables. Not surprisingly, given that one of the pathways in this mediation analysis was non-significant, the indirect effect was also not statistically significant (B = 0.0207, 95% CI = [−0.0460, 0.0005]). Thus, the present analysis provides strong evidence for a directional cascade, whereby caregivers who (at the start of the study) had higher levels of R/S were coping better over time and, consequently, reported better mental health outcomes 6 months later.

Significant pathways outside of our primary mediation question were also examined. Higher levels of coping at T1 predicted lower levels of mental health problems at T2 (like the same association across T2 and T3 in the primary mediation pathway). Furthermore, COVID-19 disruption at T1 predicted caregiver psychological distress at T2 and T3, however, it did not predict coping or R/S at any of the subsequent timepoints. One additional pathway that merits additional interpretation is the inverse relationship between T1 coping and T3 R/S.

Simple bivariate correlations revealed a *positive* association between the two variables. Similarly, caregiver coping at T2 and caregiver R/S at T3 were positively related when examined in a bivariate fashion, and in the path model. Thus, the negative association between caregiver coping at T1 and R/S at T3 is likely the result of a suppressor effect. In other words, when the very high autocorrelation (stability) of R/S is included in the model, in addition to the prediction of T2 coping, the residual variance that is captured by the T1 coping predictor is the opposite of the bivariate associations. This phenomenon is common in mediation analysis and explored further in the discussion section.

## Discussion

This study examined the relationship between caregiver religious/spiritual beliefs and practices (R/S) and mental health outcomes during the COVID-19 pandemic by exploring a potential mechanism connecting these variables (i.e., positive coping skills). Based on a longitudinal panel methodology, permitting the isolation of temporal precedence and directionality of effects, the findings revealed a longitudinal relationship between R/S and mental health (psychological distress) that is mediated by coping, even when controlling for prior levels of coping/mental health and experienced familial disruptions during the pandemic. Higher levels of R/S were associated with lower levels of psychological distress through higher levels of positive coping, suggesting that R/S is a factor that is promotive of mental health through a cognitive set of self-referential beliefs (Prime et al., 2020; Walsh, 2020), particularly about handling adversities, such as the pandemic.

Religion and spirituality conveys advantage for mental health during disasters because individuals can draw upon their beliefs and practices to make meaning out of confusing and painful moments (Walsh, 2016a,b, 2020). Especially during the pandemic, troubling questions such as, “Why is this happening to us?,” “Why is the government not able to take better actions?,” or “When will this period of stagnancy in our lives end?” are likely commonplace. These existential queries may lead to guilt if one feels like they might be responsible for negative events, or feelings of anger if they assign the responsibility to other parties such as the government (Exline et al., 2000). Neither of these scenarios are likely to be helpful to the individual (Lee et al., 2001). However, caregivers with religious or spiritual beliefs might be better able to frame such negative events as an opportunity for growth, or as a time of preparation for better things to come. Parents who are high in R/S might be able to make peace with the negative events by believing that their deity/deities has a bigger plan in place and that their current pain is simply a period of trial.

A second way R/S can promote coping and mental health is by helping cultivate feelings of hope – hope for, and faith in, the possibility of a better tomorrow, which is a vital process in the promotion of family resilience (Walsh, 2016b). Whilst excessive false hope can have the opposite effect, a healthy level of hope can inspire motivation, energy, and renewed determination to strive on (Bohart, 2002). Especially during the COVID-19 pandemic (which has lasted over a year-and-a-half at the time of this writing), individuals are likely to be experiencing burnout from the prolonged, unrelenting stream of bad news and setbacks, and a lack of change in circumstances (Prime et al., 2020). People with religious and spiritual beliefs may also possess faith that things will change soon (Chidarikire et al., 2020). For example, “that God is good, and God is kind” is a core belief within Islam and Christianity. Thus, it is possible that Muslims and Christians could be more likely to hold onto hope that their God will eventually help them. Ongoing research that pays attention to religious denomination during COVID-19 is warranted.

This study also revealed a surprising finding. In the presence of other variables in the model, caregiver coping at T1 had a negative association with caregiver R/S beliefs and practices at T3, whilst caregiver coping at T2 had a positive association with caregiver R/S beliefs and practices at T3. This could be the result of a suppressor effect. A suppressor is defined as a variable that “increases the predictive validity of another variable by its inclusion in regression analysis” (Conger, 1974, p. 36). Suppression effects can take on a few forms. They are often at play in collinear multiple regression analyses, such as cross lagged models, where an independent variable is associated with a dependent variable in the opposite direction to the bivariate association (and the logical, hypothesized direction). This is most likely the case in the regression of T3 R/S, whereby the T1 and T2 coping predictor variables are highly collinear, in addition to the R/S autocorrelation. MacKinnon et al. (2000) notes that it is difficult to fully unpack suppression as a statistical artifact (vs. substantive process) within the sample (and database) in which it was initially observed, further highlighting the importance of replicating our main study findings. Despite this likely artifact, the presented results provide compelling evidence that R/S is predictive of higher levels of positive coping and, consequently, mental health over time amongst caregivers.

Religion and spirituality is a complicated variable. Prior research has been mixed on whether R/S can promote or hinder mental health outcomes (Aten et al., 2019), which may be indicative of sample-specific findings or effect modification. By showing that positive coping skills can act as a mediator between R/S and later mental health outcomes, we are able to recognize that there is an important element of appraisal at play. The present findings imply that it is not only *whether* people have R/S resources, to call upon during times of distress and loss, that can help promote mental health, but also *how* they utilize their R/S resources. When employed in a fashion that elicits positive cognitive coping statements (e.g., “I can deal with whatever comes my way,” “having to cope with stress makes me stronger”), R/S beliefs (e.g., “I look to God for strength, support and guidance”) appear to be promotive of mental health amongst caregivers, irrespective of their COVID-19 stress.

### Clinical Implications

A major goal of therapists (and allied mental health professionals) is to support individuals and families in building positive, adaptive coping skills to call upon under times of distress. For religious and spiritual people, R/S can be one of the means of helping clients build their coping skills—a domain that is often overlooked by traditional secular health professionals. The role of a therapist is not to provide meaning to clients who are struggling with adverse events; instead, their role is to help clients find their own meaning so that they can galvanize this resource on their own in the future (Walsh, 2020). For clients that come from a religious or spiritual background, R/S can act as the client’s own framework for making meaning out of adversity. This form of religiously integrated psychotherapy (where the therapist weaves a client’s own religious beliefs into more traditional psychotherapeutic methods, such as cognitive behavioral therapy) may be more efficacious for religious clients than standard CBT alone (Pearce et al., 2015). Religiously integrated psychotherapy has been supported in research, but more randomized, longitudinal, intervention studies are required in order to gain insight into the efficacy of incorporating a client’s religion into their traditional therapy (Koenig et al., 2015). Clinicians are encouraged to educate themselves on religiously integrated psychotherapy practices in order to best serve clients within their existing transcendental beliefs systems. Practitioners and public health officials are also encouraged to recognize religious communities as sources of mental health and family strength, especially in underserved, low-income, and marginalized communities (Browne et al., 2021b).

### Limitations

One of the primary limitations of this study is that the sample was somewhat homogenous, leading to issues with generalizability. Most of the sample was Caucasian and all resided in Western countries. R/S vary greatly by country, culture, socio-economic status, and race. Future studies should recruit diverse samples or replicate studies within different cultures, while testing similar hypotheses. Second, the variables used in the analysis were all self-report, which can lead to common method variance bias (Spector et al., 2017). Studies that employ multi-method assessment could add confidence to the present conclusions. Thirdly, the primary measure of R/S was the Brief Multidimensional Measure of Religiousness and Spirituality, which does not differentiate between religiosity and spirituality, *per se*. Given recent research that has been trying to tease apart the constructs of spirituality and religiosity, future studies should try to replicate the findings of this study with measures that specifically assess religiosity or spirituality (e.g., Religious Commitment Inventory; Worthington et al., 2003). While such comprehensive instruments were not possible for employment in the current longitudinal sample (where many measures, beyond those utilized in the current study, were implemented), such tools could increase internal validity and add nuance to the study conclusions. That is, subsequent longitudinal research may answer the question of “which aspects of R/S are most salient in leading to coping skills and, accordingly, better mental health in caregivers?”. Future studies might also continue to explore the role of R/S across COVID-19, subsequent to November 2020. Lastly, while participants were drawn from four different countries (United Kingdom, United States, Canada, and Australia), differences in sample size prevented us from differentiating processes as a function of country of residence. Ongoing, large scale, cross-national samples will be instrumental in this endeavor.

### Conclusion

Findings from this study make an important contribution to the existing knowledge on how the beliefs and practices associated with R/S can act as a promotive factor and correlate with better mental health through the mediating factor of positive coping. The belief systems that R/S foster are powerful and can significantly influence the way individuals and families view and interpret negative events, including the global pandemic context. Understanding the relationship between R/S and mental health, and the possible mechanisms underlying this relationship, is important for clinical intervention, for both practitioners and clients, serving to highlight the benefit of clients’ existing belief systems. Clinicians can help their clients utilize their R/S as a source of strength, comfort, and a lens through which they can make meaning of negative events in their family’s life (Walsh, 2016b). Religious and spiritual communities are undoubtedly serving as important resources for millions during this pandemic season. Ongoing partnerships between scientific psychology and faith networks will help solidify a common language of family health promotion during the gradual, ongoing process of recovery from the COVID-19 pandemic.

## Data Availability Statement

The raw data supporting the conclusions of this article will be made available by the authors, without undue reservation.

## Ethics Statement

The studies involving human participants were reviewed and approved by Human Research Ethics Committee, University of Waterloo. The patients/participants provided their written informed consent to participate in this study.

## Author Contributions

HS was the primary author and contributor to this project, including development of the research questions, statistical analyses, and manuscript writing. DB was the lead Principal Investigator of the CRAMPED study; he leads the larger study that provided the data for this project and contributed to the idea generation, statistical analyses, and manuscript revision. LC provided the support during the idea generation phase of the project, offered the statistical support, and contributed to the manuscript revision. All authors contributed to the article and approved the submitted version.

## Conflict of Interest

The authors declare that the research was conducted in the absence of any commercial or financial relationships that could be construed as a potential conflict of interest.

## Publisher’s Note

All claims expressed in this article are solely those of the authors and do not necessarily represent those of their affiliated organizations, or those of the publisher, the editors and the reviewers. Any product that may be evaluated in this article, or claim that may be made by its manufacturer, is not guaranteed or endorsed by the publisher.
